# Hebb and Cattell: The Genesis of the Theory of Fluid and Crystallized Intelligence

**DOI:** 10.3389/fnhum.2016.00606

**Published:** 2016-12-15

**Authors:** Richard E. Brown

**Affiliations:** Department of Psychology and Neuroscience, Dalhousie UniversityHalifax, NS, Canada

**Keywords:** history of psychology, fluid intelligence, crystallized intelligence, controversy, Hebb's two types of intelligence

## Abstract

Raymond B. Cattell is credited with the development of the theory of fluid and crystallized intelligence. The genesis of this theory is, however, vague. Cattell, in different papers, stated that it was developed in 1940, 1941 or 1942. Carroll (1984, Multivariate Behavioral Research, 19, 300-306) noted the similarity of Cattell's theory to “Hebb's notion of two types of intelligence,” which was presented at the 1941 APA meeting, but the matter has been left at that. Correspondence between Cattell, Donald Hebb and George Humphrey of Queen's University, Kingston, Ontario, however, indicates that Cattell adopted Hebb's ideas of intelligence A and B and renamed them. This paper describes Hebb's two types of intelligence, and shows how Cattell used them to develop his ideas of crystallized and fluid intelligence. Hebb and Cattell exchanged a number of letters before Cattell's paper was rewritten in such a way that everyone was satisfied. This paper examines the work of Hebb and Cattell on intelligence, their correspondence, the development of the ideas of fluid and crystallized intelligence, and why Cattell (1943, p. 179) wrote that “Hebb has independently stated very clearly what constitutes two thirds of the present theory.”

## Introduction

In the 1930s and early 1940s, both Raymond B. Cattell and Donald O. Hebb were critical of the ability of the currently available tests to accurately measure intelligence and both began to develop improved tests of intelligence. Cattell was in search of a “culture-free” test of intelligence while Hebb was concerned with the development of tests of intelligence which could measure the effects of localized brain damage at different ages in patients with little schooling.

At that time, intelligence testing was fuelled by a scientific interest to quantify the intelligence of different groups of people, with a focus on special classes of individuals, such as the insane, feebleminded, deaf, and delinquent. The intelligence scales used were influenced by the work of Alfred Binet and Theodore Simon with the publication of the first Binet-Simon scale in 1905 (Binet and Simon, 1905). This “measuring scale of intelligence” was originally developed for use with school children in Paris and consisted of a series of 30 brief cognitive tests. The Binet-Simon intelligence scale and its modifications (such as the Stanford-Binet scale) were the most commonly used tests of intelligence in the 1930's (Pintner, 1931), but the Wechsler-Bellevue intelligence scale, published in 1939 (Wechsler, 1939) introduced a number of innovations in intelligence testing including a test more suitable for measuring intelligence in adults rather than children; the inclusion of both verbal and performance measures of intelligence; and the omission of memory tests (Boake, 2002).

The state of intelligence testing in America in the 1930's was reviewed by Pintner (1931), who described the intelligence tests then available and the “various fields in which mental tests have been successfully used” (p. 225). As pointed out by Pintner (1931, p. 225), “The beginnings of the intelligence testing movement were closely bound up with the study of mental deficiency and abnormality” and “much of the early work had to do with the selection of mentally deficient children.” By the 1930's intelligence testing was also used to test army recruits, employees, the deaf, the blind and the foreign-born. They were also used to examine sex and racial differences in intelligence; to detect children of superior intelligence; and to study the inheritance of intelligence (Pintner, 1931).

## Cattell's work on intelligence testing

Cattell (1940a) gave a detailed critique of the Binet test and was concerned that differences in scores on intelligence tests were due to differences in social status, special abilities distinct from intelligence, or other environmental factors. The problem that he identified was that tests of intelligence focused on acquired skills and verbal ability rather than on intelligence *per se*. Cattell was interested in developing “culture free” intelligence tests based on non-verbal skills that would define a person's general intelligence “g” (Spearman, 1904) irrespective of their social status, race, verbal ability or environmental experience. Cattell thus developed a culture-free test with seven sub-tests. The tests were designed to be non-verbal and instructions were given using a series of worked examples rather than verbal instructions.

Cattell et al. (1941) outlined seven variables which influenced intelligence or “general ability” (see Table 1) and then analyzed the effects of environmental variables such as cultural knowledge and training (practice) on each test. He then compared the scores of American-born and immigrant children on his culture-free test and three other tests of intelligence: the Terman-Merrill Revision of the Binet Test, the arithmetic section of the American Council of Education (A.C.E.) Test, and the Arthur Performance Test. Based on these studies, Cattell et al. (1941) found that the Arthur test was the most culture-free, the culture-free test was second, the Terman-Merrill-Binet test was third and the A.C.E. test fourth, and concluded that culture-free tests should be administered using people from a wide range of cultures, “primitive and otherwise.”

**Table 1:** **Seven variables which determine the scores of individuals on intelligence tests (from Cattell et al., 1941, pp. 81–82)**.

For, if we agree to use the term intelligence and to speak of a single or compound “general ability,” the variations among individuals in their test scores in an intelligence test can be regarded as depending on:
(1) Variations in the innate gene endowment which is responsible for the magnitude of this general ability, perhaps, e.g., in the genes defining the sum total of cerebral neurons. (G)
(2) Variations in environmentally (i.e., post-conceptually) produced development of the general ability. (dG)
(3) Variations in the closeness of the individual's cultural training and experience to the cultural medium in which the test is expressed. (c)
(4) Variations in familiarity with tests and test situations, test training or “test sophistication.” Several slightly different and experimentally distinguishable types of preparedness are involved here. (t)
(5) Fluctuations in the underlying general ability itself, through physiological, fatigue, and other variables. (f)
(6) Fluctuations in the effective expression or application of the ability through varying strength and direction of volition and interest. (fv)
(7) Chance errors in measurement not included in the above. (e)
Resorting to a formula, for facility in later discussions, we may say that any performance P in an intelligence test is a function of the factors in the following algebraic equation:
*P* = *G* + *dG* + *c* + *t* + *f* + *fv* + *e* + *K*, where *K* is a factor to cover special abilities.

At the 1941 APA meeting, Cattell (1941) discussed his ideas on the differences between measuring intelligence in children and adults and how differences in intelligence quotients might depend on the nature of the tests given. This was based on a paper in progress at the time, but published later (Cattell, 1943), which provides a more complete analysis of the problems in measuring adult intelligence. In this paper, Cattell described 44 different intelligence tests and proceeded to critique these for their focus on verbal material, their cultural biases and poor reliability and validity. He then considered how an adult intelligence test should be constructed in order to accurately measure general intelligence “g” without any environmental bias. He focused on two problems in the development of adult intelligence tests: the number of sub-tests to use and the influence of speed vs. accuracy as a measure of intelligence. On page 177 of this paper, Cattell (1943) discussed a third problem in developing adult intelligence tests; the problem of measuring mental capacities following brain injury, a topic that he had never mentioned in his previous papers on intelligence. Cattell then introduced the concepts of fluid and crystallized intelligence, how they differed in children and adults and how they were influenced by brain injury. His description of these concepts is as follows:

1. Adult mental capacity is of two kinds, the chief characteristics of which may be best connoted by the use of the terms “fluid” and “crystallized.”
2. Fluid ability has the character of a purely general ability to discriminate and perceive relations between any fundaments, new or old. It increases until adolescence and then slowly declines. It is associated with the action of the whole cortex. It is responsible for the intercorrelations, or general factor, found among children's tests and among the speeded or adaptation-requiring tests of adults.
3. Crystallized ability consists of discriminatory habits long established in a particular field, originally through the operation of fluid ability, but not [sic] longer requiring insightful perception for their successful operation.
4. Intelligence tests test at all ages the combined resultants of fluid and crystallized ability, but in childhood the first is predominant whereas in adult life, owing to the recession of fluid ability, the peaks of performance are determined by the crystallized abilities (Cattell, 1943, p. 178).

## The obscure origins of the theory of fluid and crystallized intelligence

Since 1943, Cattell's name has been associated with the concepts of fluid and crystallized intelligence, abbreviated Gf and Gc, respectively, and the theory has been referred to as “Cattell's theory of fluid and crystallized intelligence” (Humphreys, 1967; Undheim, 1981). In some textbooks, (see for example Smolack, 1993, pp. 86–87), the origins of the theory have been attributed to Cattell (1941). In Cattell's autobiographical writings and in those of his biographers, it is stated that Cattell developed the concepts of fluid and crystallized intelligence in 1940, 1941, or 1942. Cattell (1963, p. 2) said that “the theory of fluid and crystallized general ability was first stated 20 years ago (Cattell, 1941, 1943).” In an on-line biography (Gillis, 2007, p. 6) it is stated that “while at Clark University, he clarified his theory of fluid vs. crystallized intelligence which he presented to the 1941 APA convention.” In another biography (Cattell and Horn, 2007) it is stated that “He clarified his theory of fluid and crystallized intelligence, which he presented at the 1942 APA convention.” In his autobiographical paper, Cattell (1984, p. 160) states that “Hebb and I at the very same APA meeting put forward the theory of two forms of intelligence, he calling them A and B, and I ‘fluid’ and ‘crystallized’.” In his 1943 paper, Cattell (1943, p. 179) stated that:

Hebb (1941c, 1942) has independently stated very clearly what constitutes two thirds of the present theory, for he says that “intellectual power may be needed for the first appearance of the qualitatively superior response, but not necessarily for its persistence” (Hebb, 1942), and “in any test performance there are two factors involved, whose relative importance varies with test: one factor being the lasting changes of perceptual organization and behavior induced by the first factor during the period of growth.”

Carroll (1984) however, could not find any mention of the theory of fluid and crystallized intelligence in Cattell's writings until 1943. Carroll (1984, p. 302) states that “Cattell himself refers in several places (e.g., Cattell and Butcher, 1968, p. 18) to his 1940 paper on a culture free intelligence tests as the article in which he first put forward this theory, but a careful reading of this paper reveals no such thing.” Carroll (1984, p. 302) also points out that “In other places (e.g., Cattell, 1979, p. 4) Cattell seems to suggest that the Gf-Gc theory was first announced in an APA convention paper, but the published abstract (Cattell, 1941) gives no explicit evidence on this point.” Carroll's conclusion was that Cattell's first published use of the terms “fluid” and “crystallized” intelligences was in his paper in the *Psychological Bulletin* (Cattell, 1943). He also stated that “Cattell noted the similarity of this hypothesis to Hebb's notion of two types of intelligence; possibly this reference to Hebb was an outcome of their joint appearance at the 1941 APA symposium.” (Carroll, 1984, p. 303).

Using archival materials, this paper examines the relationship between Hebb and Cattell's ideas on intelligence and examines (1) whether Hebb influenced Cattell's development of the concepts of crystallized and fluid intelligence and (2) whether Cattell's theory of fluid and crystallized intelligence was based on Hebb's notion of intelligence A and intelligence B. The remaining sections of this paper examine Hebb's research on the measurement of intelligence in brain injured patients of different ages; the papers presented by Cattell and Hebb at the 1941 APA Meeting, and the correspondence between Cattell and Hebb which followed this meeting and led up to their publications. The paper concludes with a discussion of Hebb and Cattell's work on the measurement of intelligence after 1941.

## Hebb's dissatisfaction with the use of intelligence tests for studying brain injured patients

After completing his PhD at Harvard with Karl Lashley in 1937, Hebb obtained a research position at the Montreal Neurological Institute with Wilder Penfield. From 1937 to 1939, he tested patients who had surgery of the temporal or frontal lobes to eradicate their epileptic seizures. Hebb presented these subjects with a battery of commonly used psychological tests in order to ascertain the extent to which removal of brain tissue in different locations led to impairments in specific cognitive functions. In his first study, Hebb (1939a) studied “intelligence” in four patients following removal of their left frontal lobes. The psychometric tests were performed 5 weeks, 7 months, 4 years, and 9 years after surgery, respectively. All four patients were given the Stanford Binet Test after their surgery, but only one patient was tested before surgery. In no case did it appear that IQ was reduced after surgery.

These results led Hebb (1939a) to two conclusions. First, he determined that a range of different abilities should be tested in patients with different types of brain lesions, such as “the comparison of left frontal with right frontal lobe cases, or temporal with occipital, and so on” (Hebb, 1939a, p. 86). Second, he suggested that new intelligence tests needed to be designed for testing adult clinical patients; particularly tests which have separate measures for different abilities. He found that it was difficult to rate the intelligence of patients with sensory or motor disorders, and he could not determine the nature of higher-order cognitive deficits using the Binet test. The Binet test, when used on adult clinical patients, “fails to reveal any out-and-out loss of one capacity with good retention of others; instead, the problem seems always to be, what degree of ability does the patient retain? How does his ability on one type of activity compare with that on another?” (Hebb, 1939a, p. 86). Hebb found that all abilities were not equally affected following brain lesion, but to determine the effects of brain damage required a test which measured each ability independently, enabling a comparison of their levels in order to determine which abilities were lost and which were retained.

In his second study, Hebb (1939b) tested patient R.M., a 30-year old male, who had been given a right temporal lobectomy by Penfield at age 22 to eliminate his epileptic seizures. Hebb gave the patient a battery of psychological tests rather than a single test, and found that he had high language test scores (1.5 standard deviations above the mean, on the Stanford Binet Form L, the Thorndike Word Knowledge and Kelly-Trabue Language Completion tests), low scores on non-language tests (below the mean, on the Knox-Cube, Mare and Foal, Seguin, Porteus Maze, Kohs Block tests), and complete failure on the Feature Profile test. This was the first time that Hebb had shown differences in the results of language-based and language-independent tests of intelligence. He stated that the patients high scores on the Stanford-Binet examination “as well as his manner and conversation during interviews, might easily have been convincing evidence of unimpaired intelligence if no other tests had been given. The case is sufficient to show the disparity of two human abilities after a cerebral lesion, and to show how little a high level in one may indicate a similarly high level in the other” (Hebb, 1939b, pp. 442–443).

In terms of the clinical evaluation of intelligence in brain damaged patients, Hebb (1939b, p. 443) made the observation that “As the present case shows, however, the retention of some features of normal intellectual activity cannot show that others are also retained, and the apparent subjective clarity of a patient is no guarantee that intellect is unimpaired.” Hebb then stated it was “meaningless to speak of “intelligence” as an entity which is present or absent according to the presence or absence of certain attributes of normal intelligent behavior” in the study of brain-damaged patients (Hebb, 1939b, pp. 444–445). Hebb suggested that the concept of the level of intelligence may be useful for the analysis of normal human behavior, but for the analysis of abnormal human behavior which occurs after cerebral damage, he stated that “a separate account must be given of each of the various components making up what we regard as normal behavior. There is no question then of asking to what extent intelligence is affected by any lesion, although it may be possible, or even essential, to make quantitative determinations within qualitatively distinct systems (Lashley, 1938)” (Hebb, 1939b, pp. 444–445).

This statement shows the beginning of Hebb's theory of multiple memory systems, a theory which was decisively proven by the studies on patient H.M. (Milner, 2005). In a third paper, Hebb and Penfield (1940) reported a case study of K.M., a 27-year old male who had bilateral removal of both frontal lobes for the treatment of epilepsy. This patient was given a battery of psychonomic tests before surgery and up to four times (2, 2.5, 3.8, and 16 months) post-surgery. His Stanford-Binet Form L and M IQ rose from 83 before surgery to 94 after surgery and his McGill revised army beta exam score rose from 63 to 75. A battery of language and non-language tests was also given and he scored within the normal range in all of these. In fact, his performance was often better after the operation than it was before. Hebb and Penfield (1940, pp. 437–438) concluded that, “For the effect of lesions of the frontal lobe on human intelligence, it seems that one will have to look elsewhere than to clinical observation or ratings by intelligence tests such as are now available.” They concluded that standardized laboratory tests were inadequate to reveal the defects following frontal injury in man because of insufficient control of environmental factors and the difficulty of obtaining a good premorbid rating of ability. They suggested that by studying “learning in social situations, in adaptation to drastic environmental change or in initiative and the ability to plan and organize one's affairs may be found in the impairment that we believe must exist after large lesions of the frontal lobes.” Likewise, Hebb (1941a) found little effect of right frontal lobe lesions on Stanford Binet IQ scores of two patients.

In 1939, Hebb left the MNI to teach experimental psychology in the Philosophy Department at Queen's University in Kingston, Ontario, where he continued to develop his ideas on intelligence. The head of Hebb's department was George Humphrey, who was also interested in learning, memory and intelligence (Humphrey, 1930, 1932). In 1948, Humphrey became the first professor of experimental psychology at the University of Oxford, where he wrote his book “Thinking” (Humphrey, 1951). During 1940 and 1941 Hebb continued to work on the development of his new intelligence tests for adults at Queen's University (Hebb, 1941b) and on his ideas on the effect of brain injury at different ages on intelligence (Hebb, 1942), and began to develop a new procedure for testing intelligence in rats (Hebb and Williams, 1941).

In his address at the 1940 APA meeting, Hebb (1940) discussed the flaws in the use of adult intelligence tests such as the Stanford-Binet and Wechsler-Bellevue Scales for testing adult clinical patients. In the written (1940a, unpublished) manuscript of this talk, Hebb's ideas were put more forcefully. He stated that:

In the growing child, the various abilities mature at about the same rate; they are correlated, and the concept of a general level [of intelligence] is useful. But with disease or brain injury, there may be a dissociation of abilities. The patient may have a Superior Adult rating in vocabulary together with a seven-year-old rating in “Memory for Designs.” This is scatter as it may be detected in a composite test. But to get any clear idea of what causes scatter, of what has happened to intelligence, to describe the defect and to analyze it, the composite test is useless. One must have separate tests for separate abilities.

(Hebb, 1940, p. 1)

In this presentation, Hebb stated that there was a need for new tests to “cover a wide range of adult interests” and he referred to two new tests that he was developing, the verbal test of Adult Comprehension of attitudes, motivation, etc., and a non-verbal Picture Anomaly test (described by Hebb and Morton, 1943). Cattell's presentation at the 1940 APA Meeting was a factor analysis of the concept of socioeconomic (social) status (Cattell, 1940b).

Hebb and Cattell were thus at cross purposes in their study of intelligence. While Cattell was trying to find a single measure of intelligence, “g,” that was not contaminated by environmental variables, Hebb was arguing that for his purposes, a single intelligence score was useless. He was searching for a set of subtests that would measure different types of intelligence and could be used to assess changes in separate cognitive functions after brain injury.

## Hebb's and Cattell's papers at the 1941 APA meeting

On 5 September 1941, Hebb presented his ideas on the effects of early and late brain injury on intelligence at the APA meeting in Evanston, Illinois. Hebb's presentation at 1:50 p.m. was entitled “Clinical evidence concerning the nature of normal adult test performance.” Cattell gave a presentation at 1:15 p.m. entitled “Some theoretical issues in adult intelligence testing.” Cattell's presentation (Cattell, 1941) discussed differences in measuring intelligence between children and adults, the use of factor analyses and the notion of a general intelligence. He described the differences in the distribution of intelligence in adults and children and the advantages and disadvantages of using the Intelligence Quotient. He also discussed Thurston and Thompson's alternative methods for measuring intelligence which are described in the first two sections of his 1943 paper (Cattell, 1943).

Hebb's presentation (Hebb, 1941c) discussed the differences in effects of brain injury on adults and children and the finding that “certain kinds of abilities are less affected by late than by early injury.” While I do not have a copy of Cattell's (1941) APA presentation, I do have the typed version of Hebb's (1941c) in which he presented his ideas on Intelligence A and Intelligence B as follows:

It may be proposed that intellectual development includes two distinct things: (A) direct intellectual power, by neural maturation, and (B) the development of qualitative modifications of perception and behavior. The first factor is what reaches a peak somewhere around the beginning of adolescence, declining slowly thereafter; the second is the product of the first factor.

(Hebb, 1941c, p. 5)

This description was published in Hebb's (1942) paper on early and late brain injury (published in February 1942) in the following form “Intellectual development, therefore, involves (A) the development of direct intellectual power, by neural maturation, and (B) the establishment of routine modes of response to common problems, or of perceptual and conceptual modifications leading to qualitative modifications of behavior” (Hebb, 1942, p. 289).

## Correspondence between Cattell, Hebb and Humphrey

Based on correspondence between Cattell, Hebb, and George Humphrey, Hebb's head of department at Queen's University in Kingston, Ontario, it is now possible to show how Cattell's theory of fluid and crystallized intelligence was derived from Hebb's theory of Intelligence A and Intelligence B. Following the APA meeting, Cattell sent Hebb two typed pages of material which he was planning to add to the manuscript he was submitting to the *Psychological Bulletin* on the Measurement of Adult Intelligence. In his hand-written letter, he said that he was writing “to see if you think my statement correct and to present to you an hypothesis which developed partly as a result of our talk” (Hebb, 1941-1942, R. B. Cattell to Hebb, September 22, 1941)^1^.

The two pages that Cattell sent to Hebb for his comments include the definition of fluid and crystallized intelligence as follows:

Adult mental ability is of two kinds, which may contingently be called ‘fluid’ and ‘crystallized.’ ‘Fluid’ ability is a truly general ability to discriminate and perceive relations between any fundaments, new or old. Crystallized ability consists of discriminatory response habits built up in a particular field, through the original operation of ‘fluid’ ability, but no longer requiring a true insightful perception for their successful functioning. Intelligence tests test, at all ages, the combined action of fluid and crystallized ability, recording the level of whichever is higher.

He continued to say that: “the level of the ‘crystallized’ abilities has been largely determined by the original level of the all-round, ‘fluid’ ability. ‘Crystallized’ ability is thus a dead coral formation revealing by its outlines the limits of the original living process. Loss of any system of discriminatory habits, e.g., through brain injury, is now likely to remain more localized, i.e., to affect general ability less, and to be less remediable. For as the tide of ‘fluid’ ability recedes after adolescence the ‘crystallized’ discriminatory powers persist at a level above that at which the fluid ability is capable of rebuilding them.” (Hebb, 1941-1942, R.B. Cattell to Hebb, September 22, 1941)^1^.

This letter contains the first draft of Cattell's concepts of fluid and crystallized intelligence as they appear on pages 177–180 of Cattell (1943). On 23 September 1941, Hebb replied to Cattell and said:

The way your hypothesis is presented embarrasses me. It duplicates my own, in other wording,—the one outlined that evening in discussion with you and Crook. Do you remember asking if I meant that adult scores in some cases were only “an empty shell,” and my answering that on the contrary my hypothesis implied that they were a valid index of something important in mature intelligence? In the conversation I actually outlined much more of the hypothesis than I presented next morning at the session we both attended.

Hebb went on to say that:

As I read your MS, the hypothesis is presented as new, with a new interpretation of adult intelligence. Should it not be instead as a re-statement (a clarification and extension, if you like), of the one I have already formulated? I am not happy to take the position of claiming priority, but I have put a lot of work into the matter and believe it to be important. The MS (of the long paper, not that read at APA) has, incidentally, been shown to Humphrey, Lashley, Doll, and Werner before going to the APA. What may have led to mis-understanding is that in talking to you and Crook I went into the whole thesis of the longer MS that was prepared for publication, but in 15 min at the APA could only touch on the general outline and then deal with the changes of senescence.

Hebb concludes his letter by saying:

This whole business distresses me, and I wish you would write to let me know at once if you think this [is] claiming too much. Your points about “g” are beyond the discussion as I have it, and good; and you have put the hypothesis in simpler and more graphic language—the coral figure of speech is very nice indeed. Would you like to have a look at the carbon of my longer paper, or of the one read at A.P.A.?

(Hebb, 1941-1942, Hebb to R.B. Cattell, September 23, 1941)^1^.

Hebb showed Cattell's letter to his Head of Department, George Humphrey, and Humphrey then wrote to Cattell that “I do not think however that you should present this as your own hypothesis. It is in essence the theory on which Hebb has been experimenting all the year.” He continued to say that “Some hundreds of A.P.A. members heard Hebb put out the theory at the Evanston meeting, and I hope you will forgive me if I say that I am sure you would do your scientific reputation harm by publishing the paper in the form in which I have seen it, i.e., without giving him credit.” (Hebb, 1941-1942, G. Humphrey to R. B. Cattell, September 24, 1941)^1^.

Cattell wrote back to Hebb on 25 September 1941 and enclosed with his letter the section of his article dealing with the ideas that Hebb thought duplicated his own, “in such a way as to do justice to that debt which my formulation owes your formulation.” Cattell went on to say, “Believe me I want to give credit where credit is due, for I realize (especially after reading your articles) how much work and thought you have given to the problems involved. I hope my article now makes it perfectly clear that all the physio-psychological structure in the argument is due to you. If you think my statements need further modifying in any way please do not hesitate to say so.” He concluded that “I am quite ready to believe that the common part of the theory was already formulated in your mind before it was in mine.” (Hebb, 1941-1942, R. B. Cattell to Hebb, September 25, 1941)^1^.

Cattell also replied to Humphrey to explain how he developed his theory. He said that “I made what seemed to me a tenable, though possible premature, generalization on his data differentiating the adult abilities which do and do not deteriorate and asked in my last letter if he approved of the passage in which I described this as implicit in his writings.” Cattell went on to say that “The further theories are however quite independent, and to the best of my knowledge were neither in Hebb's paper nor in our after dinner conversation. My views were based on two pieces of evidence not in Hebb's field of research, and I had already discussed them with Thurstone and with Miles. Hebb's third source of evidence fitted in beautifully with these and completed the picture, but two thirds of the picture was already there. Naturally Hebb and I in discussion gained clarifications, but this would have happened if I had discussed the same matter with any other man of Hebb's intelligence and liking for the subject.” (Hebb, 1941-1942, R.B. Cattell to G. Humphrey, September 26, 1941)^1^.

This seems to suggest that Hebb's ideas were not unique and that any number of people would have come up with the same concepts. Indeed, Cattell suggested that Hebb had not completely worked out these ideas himself, but only hinted at them; “It seems to me that this is a case of independent but converging thinking, finally converging in a conversation with inextricable give and take. However, I wonder a little whether Hebb, in recognizing these later parts of the theory as his own, really means that he recognizes an explicit statement of what was implicit in some of the facts and notions he discussed.” (Hebb, 1941-1942, R.B. Cattell to G. Humphrey, September 26, 1941)^1^.

Finally, Cattell agreed to credit Hebb, but suggested that he was being generous in doing so: “However, I trust that the revised statement which I have sent to Hebb will be agreeable to both of us, though I confess that on re-reading it I think it goes further in acknowledging priority of parts of the theory to Hebb than my reason alone indicates that it should.” (Hebb, 1941-1942, R.B. Cattell to G. Humphrey, September 26, 1941)^1^.

Humphrey obviously showed Cattell's letter to Hebb, who wrote a long reply, outlining his discussion with Cattell in Evanston on 4 September, 1941, reviewing the contents of his APA lecture and making reference to his paper in press (Hebb, 1942). He also forwarded to Cattell a typed copy of his lecture (Hebb, 1941c), with certain sentences underlined. In this letter, Hebb was quite blunt. He said that:

Your letter to Humphrey, and your MS, suggest that you must have forgotten part of the content of the paper I read at APA. Your letter to Humphrey wonders “whether Hebb, in recognizing these later parts of the theory as his own, really means that he recognizes an explicit statement of what was implicit in some of the facts and notions he discussed.” Humphrey did not write at my request; he recognized what has been explicitly (not implicitly) formulated in my MS which he had seen before the APA meeting.

Hebb went on to explain that he had, in fact, clearly worked out his theory, discussed this theory and written it into his paper in press. He said:

Actually, the hypothesis was formulated and discussed in detail in the original MS, and I discussed this with you and Crook on Thursday evening. I can understand perfectly that I must have appeared to be very greedy, if you thought that I had not formulated the idea clearly before talking to you. But this is the second point of fact. Your letters speak of my clarifying my ideas in conversation with you, but I have not touched the MS since apart from meeting some criticisms of my clinical data, made by Dr Doll and Dr Heinz Werner, who received the MS last June.

With his letter, Hebb sent the original copy of his typed out APA presentation and underlined the sections, which Cattell had used in the new section of his paper. Hebb said that:

The essential point of fact, which led to my writing to you in the first place, is that I had publicly presented (though not, in 15 min, with a discussion of all its implications) an hypothesis which was identical (except for wording) with part of your hypothesis. You told me in your first letter that your theory had been conceived as a result of our conversation; it was clear to me at the time that you were not thinking in the terms that I was; you told me the next morning, after I had read my paper and sat down beside you, that my paper had given you some ideas and that you would write to me about them; and it was a shock to me later to meet my own hypothesis in your MS. [emphasis added] I felt consequently that you had, in perfectly good faith, only later realized the full implications of my paper and of our earlier conversation, and had not remembered how much of those implications I had stated explicitly. I know to my cost how easy it is to do something of the kind. But I had stated the hypothesis, twice, - once in conversation and once publicly.

Hebb concluded by asking Cattell to look at the evidence for himself, “I beg you not to take these statements on faith, but to verify them by the two MS's.” (Hebb, 1941-1942, Hebb to R.B. Cattell, September 30, 1941)^1^.

Hebb sent the typed version of his 1941 APA talk to Cattell. The top of the typescript (Hebb, 1941c) has the hand written comment “RBC please note what is underlined on pp. 4–6.” These underlined sections are as follows:

My thesis here is that intellectual power may be needed for the first appearance of the qualitatively superior response, but not necessary for its persistence. It may be proposed that intellectual development includes two distinct things: (A) direct intellectual power, by neural maturation, and (B) the development of qualitative modifications of perception and behavior. The first factor is what reaches a peak somewhere around the beginning of adolescence, declining slowly thereafter; the second is the product of the first factor. It means lasting changes in the way one sees things, the way one approaches a problem.

- Hebb (1941c, p. 5)

Part of what we mean by intelligence in an adult, therefore, may consist of a store of solutions, so to speak, for common problems—points of view, methods of approach, ways of seeing things. Although they are properly a product of intellectual power, they are an important part of what we call intelligence. The decline of intellectual power from the age of 16 or thereabouts does not mean that the subject at once begins to be less intelligent.

- Hebb (1941c, p. 6)

Cattell wrote back to Hebb on 8 October 1941, and stated that:

The articles which I have received, and which I have perused with much interest, seem to me to make finally possible a complete clarification of the situation. It is evident that you had quite explicitly stated what amounts to about two-thirds of the generalizations listed in my hypothesis, and I should be glad if you would tell Humphrey that I retract the notion that I may have made explicit what was implicit in your discussion.

(Hebb, 1941-1942, R.B. Cattell to Hebb, October 8, 1941)^1^

Cattell rewrote the new section of his paper to indicate that Hebb had already developed a similar theory and to indicate how his own ideas differed from those of Hebb. Cattell also enclosed a revised version of his manuscript which Hebb edited. On 11 October 1941, Hebb sent his final changes to Cattell and stated that: “Your letter was a great relief to me, by indicating that we are out of the mess. I look forward to clearing up any minor points over a glass of beer at the next APA meeting.” Hebb suggested a change to Cattell's new section, “The only change I would suggest is that in your listing of the points on which you and I differ (toward the bottom of the third page of your MS) you put more stress on at least one of your contributions: until I talked with you I could find no basis for reconciling “g” with clinical data and with effect of age on test score patterns; so you have done more than to identify the first factor with “g.” I would have suggested something like:

The present theory differs from Hebb's (1) in identifying the first factor with ‘g’. Thus, modifying the ‘g’ hypothesis somewhat, but at the same time providing a basis for accounting in terms of ‘g’ and ‘s’ for the effect of age, brain injury or brain disease upon test scores. (2)…”

(Hebb, 1941-1942, Hebb to R. B. Cattell, October 11, 1941)^1^.

On 15 October 1941, Hebb again wrote to Cattell to say that he was adding a footnote to his paper (Hebb, 1942, p. 290) to refer to Cattell's work. In this letter, he asked Cattell “Would you let me know if the following statement seems acceptable to you for inclusion in my paper? Its context is in referring to the desirability of finding some common ground between the qualitative analysis, by quantitative methods, of Thurstone, and the clinical analysis of my MS.” The statement was as follows:

… Cattell (1943) in an independent approach to the problem has also shown that Spearman's ‘g’ can be related to this type of analysis. His theory was developed in part on the basis of the present discussion, but his treatment is based on a wider range of facts and has significantly added to the theory by showing among other things that it makes the ‘g’ hypothesis (with some modification) consistent with facts from the clinical and normal adult fields which would otherwise seem incompatible with it.

(Hebb, 1941-1942, Hebb to R. B. Cattell, October 15, 1941)^1^.

Cattell replied on 18 October 1941, and said “I want to thank you for your remarks in the first letter…and to say that your proposed statement as set out in your second letter seems to me unobjectionable. My article is being published under the title THE MEASUREMENT OF ADULT INTELLIGENCE in the Bulletin.” (Hebb, 1941-1942, R.B. Cattell to Hebb, October 18, 1941)^1^.

## Cattell's published paper

In the published version of his paper, Cattell (1943, p. 179) states that “Hebb (1941c, 1942) has independently stated very clearly what constitutes two thirds of the present theory, for he says that “intellectual power may be needed for the first appearance of the qualitatively superior response, but not necessarily for its persistence” (1942), and “in any test performance there are two factors involved, whose relative importance varies with the test: one factor being the lasting changes of perceptual organization and behavior induced by the first factor during the period of growth.” Cattell also added the statement that he and Hebb had agreed on, which stated the four ways that his theory differed from Hebb's theory. He said:

The present theory differs from Hebb's: (1) in identifying the first factor with “g” among children, thereby modifying Spearman's “g” hypothesis to take account of findings regarding age changes and brain lesions; (2) in considering intellectual development to be a continuous increase in the capacity to perceive hierarchically more complex relations rather than an appearance of new, qualitatively superior responses; (3) in supposing, contrary to Hebb (1942) and Lashley (1938), that the high intercorrelation of tests in childhood is due to a functional unity of fluid ability and therefore, presumably, of cerebral action and is not an artifact arising from pre-established harmony in growth; (4) in connecting with more connotations, thereby making the theory more rigid, more remote from the level of a descriptive hypothesis, more subsumptive of data from different fields.

(Cattell, 1943, pp. 179–180)

Based on the correspondence summarized above, it is clear that the theory of two types of intelligence was developed by Donald Hebb and incorporated by Cattell after he heard Hebb describe his theory at the 1941 APA meeting in Evanston, Illinois. It would appear that Hebb's theory of intelligence A and intelligence B was renamed by Cattell and incorporated into his 1943 paper. It is thus clear that the ideas of fluid and crystallized intelligence were Hebb's and were adapted by Cattell. In fact, Hebb edited some of the sections of Cattell's 1943 paper.

In his later writings, however, Cattell maintained that he developed his theory of fluid and crystallized intelligence independently from Hebb. Cattell (1984, p. 160), for example, states that, “[E.G.] Boring, either from pride of Harvard (where I then taught) or from his sense of history, wrote letters to clear up possible plagiarism. There was none: we had independently reached the same position, Hebb through physiology, I through factor analysis.” Cattell's student Horn (1966, p. 554) also felt that Cattell's theory was distinct from Hebb's. He stated that, “At a purely theoretical-verbal level, the concepts here have some resemblances to Hebb's notions about general abilities labeled simply *A* and *B*. But unlike Hebb's theory, wherein ability *A* is an unmeasured and unmeasurable (in behavioral terms) physiological potential, the fluid-crystallized theory specifies definite and distinct behavioral referents for the two major concepts.”

## Hebb's (1942) paper and why it was important

Hebb's (1942) paper was an important step in the development of his ideas on intelligence and brain function. In this paper he made six points. The first was that “brain injury affects test scores unequally.” To provide evidence for this, Hebb reviewed case studies and compared results of different lesions on verbal and non-verbal tests. Hebb explained that the retention of some abilities at near normal levels after large amounts of cerebral cortex were removed were of interest because there was a significant loss of other abilities. This was the beginning of Hebb's ideas of multiple memory systems. Second, he pointed out “the usefulness of an experimental test battery” using a number of verbal and non-verbal tasks to assess the selective effects of brain damage. Hebb described two psychometric patterns following brain injury in adults, “the non-aphasic syndrome, with vocabulary, in particular, high and other abilities low, and the aphasic syndrome, with non-verbal abilities markedly higher than verbal abilities” (p. 283). Third, he dissociated the effects of early brain injury “birth injury” from adult brain injury in that both verbal and non-verbal abilities were reduced by early brain injury. In contrast to Cattell (1943), Hebb's hypothesis was the opposite of that proposed by Lashley (1938) who found fewer effects of early as opposed to late brain injury. Fourth, Hebb proposed that the development of intelligence and the retention of intelligence were qualitatively different processes and proposed that “learning to solve a problem demands more intellectual effort than solving more problems of the same kind” (p. 286). Fifth, to account for the differential effects of early and late brain lesions, Hebb (1942, p. 287) proposed the existence of two factors involved in intelligence which affect performance on intelligence tests: “one factor being present intellectual power, of the kind essential to normal intellectual development; the other being the lasting changes of perceptual organization and behavior induced by the first factor during the period of development. Roughly, the one concerns power of “reasoning,” of synthesis and invention; the other skill (that is, a factor due to experience).” He concluded by saying “The contrast is not between intelligence and knowledge, but between capacity to develop new patterns of response and the functioning of those already developed.”

Sixth, this proposal led Hebb to the hypothesis that intelligence has two components: “Intellectual development, therefore, involves (*A*) the development of direct intellectual power, by neural maturation, and (*B*) the establishment of routine modes of response to common problems, or of perceptual and conceptual modifications leading to qualitative modifications of behavior” (p. 289). Thus, in this paper, Hebb laid the groundwork for his later ideas on the synapse, the cell assembly and the cortical organization underlying behavior (Hebb, 1949).

## What happened after 1941?

In 1942, Hebb moved to the Yerkes Primate Centre in Florida to work on chimpanzees with Lashley, but he did not abandon his work on brain damage and intelligence. He wrote a critical review of the literature on the effects of damage to the frontal lobes (Hebb, 1945) and continued to work on the measurement of adult intelligence (Hebb and Morton, 1944). He also developed new tests to measure verbal and non-verbal aspects of intelligence (Hebb and Morton, 1943).

The development of verbal and non-verbal tests of intelligence for adults was Hebb's attempt to dissociate the aphasic and non-aphasic syndromes following adult brain injury (Hebb, 1942). These new tests were based on Hebb's ideas that there were two components to intelligence, one of which is “innate potential” and other is the result of experience, and that a single intelligence score was not sufficient to measure the range of abilities of a normal adult; what was needed was “to measure independently as varied a number of achievements as possible” (Hebb and Morton, 1944, p. 221). Hebb and Morton (1944) point out that taking a number of independent measures of intellectual performance “reduces the danger of mistaking environmental deprivation for innate defect.” Hebb and Morton (1944) were also critical of the use of IQ tests, designed to test the development of children's intellectual ability, for measuring adult intelligence, and stated that “there is a serious need of a new test material suited to adult work” (p. 222), which was the purpose of the development of the new adult comprehension tests by Hebb and Morton (1943).

Hebb then applied his ideas on the two aspects of intelligence to animal studies. He published his procedure for rating animal intelligence, the so-called Hebb-Williams maze, which had been developed in 1941 at Queen's University (Hebb and Williams, 1946) and used this maze to test rats that had been reared in an enriched home environment vs. rats reared in laboratory cages (Hebb, 1947, 1949). This experiment showed that measures of intelligence could be altered significantly by environmental experience, and was the first experimental evidence that intelligence A and B could be dissociated. Later, Hebb's students made improvements to the Hebb-Williams maze as a test of intelligence for the rat (Rabinovitch and Rosvold, 1951), and used this test to examine the effects of environmental enrichment on problem solving in rats (Forgays and Forgays, 1952; Hymovitch, 1952). Hebb's students also showed that there was an interaction between brain damage and environmental experience on intelligence in rats (Lansdell, 1953; Smith, 1959), indicating that the physiological and environmental aspects of intelligence could be dissociated. Later, Hebb's students began to examine the effects of environmental enrichment and deprivation on measures of intelligence in dogs (Thompson and Heron, 1954).

As noted by Horn (1966, p. 554), Cattell did not do any follow-up research on the theory of fluid and crystallized intelligence, but “left it on the shelf” until 1963, when he showed that two general ability factors could be derived from factoring culturally embedded and culture fair intelligence test results from a sample of 277 children in the 7th and 8th grades (Cattell, 1963). In this paper he concluded that one of these factors “fits the crystallized ability factor measured in traditional intelligence tests and the other a fluid general ability measured in culture-fair intelligence tests” (p. 20). Cattell's student John Horn continued to develop the theory of crystallized and fluid intelligence (Horn and Cattell, 1966, 1967).

These theories of intelligence and the many tests developed by Cattell during his academic career were, unbeknownst to many in the field, used as an important source of empirical support for his radical socio-religious beliefs. Cattell's philosophy, eventually termed Beyondism (Cattell, 1972, 1987; Mehler, 1997), was meant to replace Christianity with an evolutionary based religion that promoted evolutionary progress through group competition to ensure that only the fittest groups would survive. According to Cattell, this could be accomplished by scientifically distinguishing and segregating the population by race, and then implementing eugenics programs that would increase the general intelligence of the population (Cattell, 1933). These ideas were first developed in the 1930's (Cattell, 1933, 1937, 1938), but remained consistent over his academic career (Cattell, 1950, 1972, 1987). In fact, it is these extreme socio-religious beliefs and their relationship to his scientific contributions that lead to the postponement of the presentation of the American Psychological Foundation Gold Medal Award for Life Achievement in Psychological Science to Cattell in 1997. The selection of Cattell for this award, occurring months before his death at 92 years of age, ignited a storm of controversy among the scientific community about Cattell's long-documented history of racial segregation and eugenics. In the end, Cattell decided to withdraw his name from consideration for the award and wrote an open letter to the American Psychological Association defending himself and his work by saying that his statements were misinterpreted and taken out of context (Cattell, 1997; see Tucker, 2009).

## Conclusions

The information cited in this paper leads to the conclusion that “Cattell's” theory of fluid and crystallized intelligence is Hebb's theory of Intelligence A and Intelligence B, given another name and popularized by Cattell. Cattell's theory was Hebb's idea. My search for Hebb's role in the genesis of the concepts of fluid and crystallized intelligence was stimulated by a letter from Dr. Bem Allen of Western Illinois University (15 September 2009), who stated that “I think …that Cattell originated the labels ‘fluid’ and ‘crystallized,’ though I have never found where he first introduced these labels. However, I examined the 1940 and 1942 APA convention presentation titles and abstracts in Psychological Bulletin for the years that Cattell, Heather Cattell and John Horn claimed that Cattell first reported on two kinds of intelligence.” He continued to say that “I found no presentation by Cattell that had anything to do with intelligence, but Hebb presented on two kinds of intelligence in 1940. Also, in 1942 Hebb published a paper in the Proceedings of the American Philosophical Society that very clearly dealt with two kinds of intelligence, one fitting the fluid mold and the other basically identical to “crystallized” intelligence (he used terms akin to problem solving and knowledge).”

Dr. Allen concluded his letter by saying that “The two kinds of intelligence are a very important contribution. If Gc and Gf should be credited to Hebb, it would extend the life of his impact on psychology and that would be good for our discipline.” (Personal communication from B. Allen to R. E. Brown, September 15, 2009). This is discussed in his paper on Cattell (Allen, 2010).

From all of the evidence cited above it can be concluded that Hebb's theory of intelligence was adopted and extended by Cattell, who renamed Hebb's intelligence A and intelligence B “fluid and crystallized intelligence.” “Intelligence A and B, when expressed together with the definitions provided by Hebb were scientifically valid, but who can remember which is which? By calling these fluid and crystallized Cattell provided names that, via compelling metaphor, cemented the concepts in the minds of readers and listeners” (Personal communication from R. M. Klein to R. E. Brown, November 15, 2016). Part 3 of Cattell's (1943) paper was based on Hebb's 1941 APA paper (Hebb, 1941c) and the ensuing discussion. Hebb pointed this out to Cattell, who claimed that he derived the theory from Hebb's implicit comments. Hebb sent Cattell the typescript of his lecture and 1942 paper in press, to show that he had explicitly stated his ideas. Cattell added a statement to his 1943 paper that Hebb and he had independently come up with the same idea. Hebb helped edit the section of Cattell's paper that showed how Cattell's ideas differed from his own. Despite Cattell's feeling that he gave Hebb more credit than he was due, Hebb was overly gracious in not insisting that he had developed the ideas before Cattell. The theory of fluid and crystallized intelligence therefore, should be called the Hebb-Cattell Theory.

## Author contributions

The author confirms being the sole contributor of this work and approved it for publication.

## Funding

This research was funded by NSERC of Canada.

### Conflict of interest statement

The author declares that the research was conducted in the absence of any commercial or financial relationships that could be construed as a potential conflict of interest. The reviewer RK declared a shared affiliation, though no other collaboration, with the author RB to the handling Editor, who ensured that the process nevertheless met the standards of a fair and objective review.

## Footnotes

^1^All of the correspondence between Hebb, Cattell and Humphrey quoted in this paper is held in the McGill University Archives, Donald O. Hebb papers (MG1045) in Montreal, Quebec.
